# Experiences of Patients and Therapists Testing a Virtual Reality Exposure App for Symptoms of Claustrophobia: Mixed Methods Study

**DOI:** 10.2196/40056

**Published:** 2022-12-05

**Authors:** Gwendolyn Mayer, Nadine Gronewold, Kirsten Polte, Svenja Hummel, Joshua Barniske, Jakob J Korbel, Rüdiger Zarnekow, Jobst-Hendrik Schultz

**Affiliations:** 1 Department of General Internal Medicine and Psychosomatics Heidelberg University Hospital Heidelberg Germany; 2 Information and Communication Management Technische Universität Berlin Berlin Germany

**Keywords:** virtual reality, exposure therapy, anxiety disorders, claustrophobia, think-aloud, mixed methods, virtual reality exposure therapy, VR, anxiety, therapy, mental health, user experience, perspective

## Abstract

**Background:**

The effectiveness of virtual reality exposure (VRE) in the treatment of anxiety disorders is well established. Several psychological mechanisms of VRE have been identified, whereby both emotional processing and the sense of presence play a key role. However, there are only few studies that contribute to our knowledge of examples of implementation in the case of VRE for claustrophobia based on patients' experiences and the perspective of therapists.

**Objective:**

This study asks for key elements of a VRE app that are necessary for effective exposure for people with claustrophobic symptoms.

**Methods:**

A mixed methods design was applied in which patients (n=15) and therapeutic experts (n=15) tested a VRE intervention of an elevator ride at 5 intensity levels. Intensity was varied by elevator size, duration of the elevator ride, and presence of virtual humans. Quantitative measures examined self-reported presence with the Igroup Presence Questionnaire (IPQ) ranging from 0 to 6 and 15 Likert-scaled evaluation items that had been developed for the purpose of this study, ranging from 1 to 5. In both measures, higher scores indicate higher levels of presence or agreement. Think-aloud protocols of the patients and semistructured interviews posttreatment of all participants were conducted to gain in-depth perspectives on emotional processes.

**Results:**

The intervention induced a feeling of presence in patients and experts, posttreatment scores showed a high IPQ presence score (mean 3.84, SD 0.88), with its subscores IPQ spatial presence (mean 4.53, SD 1.06), IPQ involvement (mean 3.83, SD 1.22), and IPQ experienced realism (mean 2.75, SD 1.02). Patients preferred a setting in the presence of a therapist (mean 4.13, SD 0.83) more than the experts did (mean 3.33, SD 1.54). Think-aloud protocols of the patients revealed that presence and anxiety both were achieved. Qualitative interviews of patients and experts uncovered 8 topics: feelings and emotions, personal story, telepresence, potential therapeutic effects, barriers, conditions and requirements, future prospects, and realization. The intensity levels were felt to appropriately increase in challenge, with ambivalent results regarding the final level. Virtual humans contributed to feelings of fear.

**Conclusions:**

Key elements of a VRE app for claustrophobic symptoms should include variation of intensity by adding challenging cues in order to evoke presence and anxiety. Virtual humans are a suitable possibility to make the intervention realistic and to provide a sense of closeness; however, some of the fears might then be related to symptoms of social phobia or agoraphobia. Patients may need the physical presence of a therapist, though not all of them share this view. A higher degree of sophistication in the intensity levels is needed to deliver targeted help for specific symptoms of anxiety.

## Introduction

### Virtual Reality Exposure in the Treatment of Anxiety Disorders

Anxiety disorders are the most prevalent mental disorders worldwide [1], with a considerable impact on the individual's quality of life [2] and on occupational outcomes [3]. A substantial share of patients with anxiety show comorbid depressive symptoms and remain chronically affected [4]. Treatment guidelines recommend cognitive behavioral therapy (CBT) as an effective treatment [5-7], of which exposure therapy (ET) is a core element of evidence-based approaches [8]. This specific therapy form builds upon the mechanisms of conditioning and learning. By exposing the patient to the feared situation or object, they may overcome the anxiety. Mechanisms behind this therapeutic effect have been identified as cognitive and emotional factors, such as inhibitory learning, emotional processing, and self-efficacy [9]. ET has been elaborated in various formats beyond the classical in vivo exposure, including the computer-based presentation of the feared stimulus. Digital technology was formerly used to create an exposure situation based on videos or images. More recent approaches use augmented reality (AR) and virtual reality (VR) apps with the use of mobile technology and head-mounted displays (HMDs), respectively [10]. Virtual exposure has the advantage of increasing the self-reflectiveness of patients [11] and provide a better level of standardization for clinicians [12].

The state of the art of virtual reality exposure (VRE) for anxiety disorders allows for multiple purposes in terms of objectives and scope of diagnoses. VRE is currently used for the assessment and treatment of anxiety and related disorders, such as obsessive-compulsive disorders and posttraumatic stress disorders [9,13], as well as a diagnostic tool for paranoid ideations [14]. Studies show promising results for the treatment of social anxiety disorders and fear of public speaking [15,16] by including virtual humans that may be controlled by a therapist [17]. A comparable effectiveness of VRE in the treatment of anxiety disorders compared to in vivo treatment has been successfully identified in the broad spectrum of anxiety-related disorders [18,19] and specific phobias [20,21]. Deterioration rates are likely to occur randomly and could not be traced back to the application of VRE therapy in a meta-analysis with several treatment approaches for anxiety [22].

### Psychological Mechanisms of VRE in Specific Phobias

The effectiveness of ET for specific phobias in general can be explained by several factors. In early studies, the emotional processing theory by Foa and Kozak [23] served as an explanation [9]. Foa and Kozak [23] showed that in exposure, (1) the fear response has to be activated, then (2) a habituation (decrease) of fear will take place gradually, and (3) the initial reaction to the feared situation or object will decrease over several sessions. In line with these findings, the avoidance of relaxation during exposure has been detected to be an important emotional prerequisite for a successful ET in several studies, as compiled by Böhnlein et al [10]. In their review, they found several other emotional, cognitive, and behavioral factors that explain the success of ET (eg, cognitive factors as self-efficacy before and the focus on changes in fear-relevant cognitions during the exposure), that is, by increasing awareness for cognitions before and after experiencing stress during ET. An important behavioral success factor is the variation of the context or stimulus variation [10]. With respect to the latter, different levels of stimuli variations seem to be beneficial for the reduction in phobic symptoms. For this reason, VRE apps usually offer different intensity levels by gradually adding more fear-evoking cues within the virtual environment (see, eg, Ref. [24] for social phobias and Ref. [25] for acrophobia).

When working with VR environments, the feared context or stimulus can only be felt by the user if there is a sense of presence, in other words a feeling of “being there,” during the VR session [26]. Three aspects constitute the nature of presence, which are (1) spatial presence (ie, the degree of experiencing the environment as a 3D room to interact in), (2) involvement (ie, the strength of internal focus toward the VR environment and the degree of “forgetting” the real world), and (3) realness (ie, the degree of comparability of the VR environment with the real world) [27]. Study results show a positive relationship between the sense of presence and anxiety, although with a larger effect for fear of animals and fear of flying and nearly no effect for social phobias and claustrophobia [26]. Presence is closely related to phobic elements within the VR environment and therefore to a feeling of anxiety, but presence alone is not sufficient for a positive treatment outcome [28]. Presence is a concept that describes the perspective of the user, while the objective attribute of the technology enabling the user to feel presence is summarized by the term “immersion” [29]. In anxiety treatment, higher levels of immersion are associated with the correlation of presence and anxiety [26].

Nevertheless, presence seems to be no necessary requirement for inducing fears within VR, as shown in a study that investigated whether social anxiety could be induced more effectively by in vivo talking to somebody or during conversation with avatars. The authors emphasized that although virtual conversation was rated with lower presence, participants reported a higher degree of fear toward their virtual counterpart [30].

### Relevance and Examples of the Treatment of Claustrophobia

Claustrophobia belongs to the category of several specific phobias and refers to an irrational fear and avoidance of enclosed places (elevators, tunnels, caves) and the inability to escape from them. The fear seems to be composed of a fear of suffocation and of confinement [31]. Epidemiological studies estimate a lifetime prevalence of 2.2%, with a treatment rate of 27.5% [32]. However, subclinical symptoms in daily life that are reported by the general population have been observed in 12.5% [33], and more low-threshold treatment offers might be necessary to meet the demands of these people. Specific phobias are associated with impairment and show high comorbidity with other mental health disorders [32]. Moreover, a recent population-based survey in 24 countries showed that patients suffering from specific phobias who were seeking professional help received helpful treatment only in 23% of cases from their first professional contact [34]. In the case of claustrophobia, the limitations due to this phobia become highly relevant for patients preparing for an MRI scan. Many radiographers report being confronted daily with claustrophobic patients [35]. VR technology is currently used for symptom reduction in MRI scans in educational programs [36,37] or for the purpose of distraction [38].

Only few studies describe VRE specifically aiming at the treatment of claustrophobia, although the first studies were conducted based on a case study as early as 1998 [39] and by the same author with 4 participants in 2000 [40]. As reported by Ling et al [26], the role of presence in VRE for claustrophobia was investigated only in 2 studies, which did not find a relationship between presence and the degree of anxiety felt. Following the above-mentioned review of Böhnlein et al [10], important behavioral factors might have been missing, such as sufficient variation of context, which is a necessary success factor of ET. Other studies make use of rather simple settings to induce claustrophobic fears (eg, a closed box [41] or a room with a sudden fire), which might trigger confounding fears, in addition to claustrophobia [42]. The experience of narrow space in reality is not only influenced by the size of a room but also by the presence of other people, such as in crowded places or supermarkets. This is especially the case in an elevator, where one has to share a room that is already narrow due to the presence of others. This might be one reason some authors consider claustrophobia a prodromal stage of agoraphobia [43].

### The Therapeutic Experts' Perspective on VRE

Former study results show a generally high acceptance of VRE among therapists but at the same time a low familiarity [44]. Studies so far have analyzed the experiences and treatment effects on patients. However, we did not find any study that included the experiences made by therapists of different therapeutic backgrounds testing the same intervention as their potential patients, even in studies where the VR sessions were therapist-led by voiceover instructions and therapists thereby stayed in contact with their patients [45]. The perspective of therapeutic experts is essential to understand the experiences reported by patients during and after VRE and different views due to the variety of approaches (CBT or psychodynamic) might add valuable insights. For the implementation in anxiety treatment, it is also interesting to find out which factors might influence the therapists' attitudes and openness toward the use of VRE interventions in their therapies. As therapeutic experts will prescribe this kind of intervention in the future, understanding their perspective is crucial for future developments.

### Objectives

The aim of this study was to test a VR app with different intensity levels for the future use of VRE for claustrophobic symptoms in order to find key elements that are necessary for exposure. To reach this goal, we asked whether our fully immersive intervention is sufficiently able to induce feelings of presence as a basic requirement to evoke anxiety. Furthermore, the following question was studied: Did intensity levels reflect an appropriate growth in challenge for future personalization of the app?

Additionally, we were also interested in the perspective of psychotherapeutic experts of different approaches regarding the content of the intensity levels and potential barriers and facilitators for implementation into their future clinical practice.

## Methods

### Study Design

This study was a nonrandomized feasibility study that analyzed the experiences and perspectives of patients and therapeutic experts regarding a VRE app for symptoms of claustrophobia.

The app was developed by clinicians and technology experts in the context of the nationally funded project SELFPASS (Self-administered Psycho-Therapy Systems) that has been described elsewhere [46,47]. The design and content of the intervention were closely oriented to psychotherapeutic manuals of CBT [48]. The intervention was designed as a fully immersive system with the use of HMD technology that has the advantage of shutting out physical reality, while providing a high degree of fidelity [49].

A convergent mixed methods approach was used, with quantitative measures pre- and posttreatment, think-aloud protocols during treatment, and qualitative interviews posttreatment [50]. This combination of methods allowed for the integration of generalizable aspects with quantitative scales, on the one hand, and a detailed description of individual experiences with qualitative material, on the other hand [51]. The latter had an impact on the sample size considerations. As we chose to carry out qualitative interviews and to analyze think-aloud protocols, we limited the group size to 15 participants in each group. We chose equal group sizes to be able to compare scales. However, as emphasized by Malterud et al [52], the more information power the sample holds, the lower the sample size that can be planned. One important factor that contributes to information power is the quality of dialogue that we considered as high due to the close contact to the participants during and after the intervention.

### Recruitment and Procedures

Recruitment was performed by postings and mail distribution services to patients, employees, and experts within the Heidelberg University Hospital’s Department of Internal Medicine and Psychosomatics in the Medical Faculty. The criteria for inclusion were a minimum age of 18 years and the capacity to provide consent. Patients were included when they reported suffering from symptoms of claustrophobia and when they had received a diagnosis of an anxiety disorder of any kind by a physician at the Heidelberg University Hospital within the past year. A Structured Clinical Interview for DSM-5 (SCID) interview [53] was then conducted by author NG, who is a trained psychotherapist. This interview was conducted to exclude those patients in danger of suicidality or psychosis and to confirm the self-reported symptom profile. Experts were considered eligible if they held a university degree in medicine or psychology and were active in the field of psychotherapy, psychiatry, or psychosomatics. To obtain broad perspective of feedback, experts from various psychotherapeutic approaches were considered eligible, such as psychodynamic, psychoanalytic, and systemic approaches, as well as CBT.

The testing procedures were carried out in a separate room within the facilities of the hospital. For the time of the study, an area of at least 9 m^2^ was reserved. The hardware consisted of an HTV VIVE Pro Eye headset, 2 base stations, and 2 controllers. The 2 base stations were set up in the corners of the room to serve as reference points for the headset and controllers. Participants' safety was guaranteed by the presence of at least 1 researcher, who took care for the position of the cable. Hygienic measures were applied before and after testing to reduce the risk of infection during the COVID-19 pandemic.

### Ethical Considerations

Ethical approval was obtained from the Ethics Committee of the University of Heidelberg (S-746/2020), and recruitment started thereafter. All participants signed an informed consent form.

### Measurements

Participants completed a short questionnaire with demographic details, the technology commitment scale (TCS) [54], and the State-Anxiety Scale of the State-Trait Anxiety Inventory (STAI-S) [55]. The concept of technology commitment refers to the individual readiness to use technology, which is based on 3 underlying concepts: technology acceptance, technology competence conviction, and technology control conviction. All 12 items range from 1 (fully disagree) to 5 (fully agree), with single items to be coded reversely. The instrument shows high correlation to the concept of self-efficacy; however, it is more closely related to the use of technology [56]. There are no cut-off values for the TCS; however, population studies have shown mean values of mean 3.73 (SD 0.62) in technology commitment, a mean of 3.27 (SD 0.94) for technology acceptance, a mean of 4.16 (SD 0.80) for technology competence conviction, and a mean of 3.75 (SD 0.74) for technology control conviction [56]. We expected no differences in technology commitment between patients and experts, as both groups should be willing to engage with the VRE. The STAI-S was applied pretreatment in order to validate the anxiety of patients before the intervention that should be higher than in the other group. A cut-off of 41 is recommended in the literature to differentiate between healthy and clinical levels of anxiety [57]. We expected higher scores in the STAI-S for the patients.

Thereafter, they started the virtual tasks. After completion of at least 3 tasks, they filled out the Igroup Presence Questionnaire (IPQ) that measured the subscales spatial presence, involvement, experienced realism, and general presence on a 7-point Likert scale with 14 items, with higher results indicating a higher sense of presence [58]. Although the IPQ is a validated instrument, the authors present no norm values; however, studies by the authors with values ranging from 0 to 6 were interpreted as relevant when exceeding a mean of 3 during the intervention [59]. As the intervention was designed as a fully immersive app, we expected high levels of presence, as measured by the subscales of the IPQ in both groups.

A set of 15 evaluation items was developed for the purpose of this study. These items were Likert-scaled with a range from 1 (lowest) to 5 (highest). The first 15 items asked about the appropriateness of the design, its clarity, the length of the intervention, and the perceived usefulness. Two further items asked for the possibility to carry out the intervention in the future while being alone or whether there is a need of a therapist being present. We asked these questions to gain insights into the different perspectives; however, group comparisons were not relevant.

All participants were invited for 1 session of a duration of 60-90 minutes. They were asked to complete at least 3 tasks during the session. The experts were asked to complete tasks 1, 3, and 5. However, every participant could perform all tasks by choice.

The participants were invited to speak aloud during the intervention and to talk frankly about their observations, feelings, and emotions, if they wanted to do this. They were reminded to express their thoughts. However, some participants were too involved to make use of this opportunity and forgot to do so. This so-called think-aloud method was applied to obtain immediate insights into the experiences of the participants [60]. As our analysis focused on the experiences of patients carrying out VRE, we only reported on the results of the think-aloud protocols of the patients.

**Figure 1:**
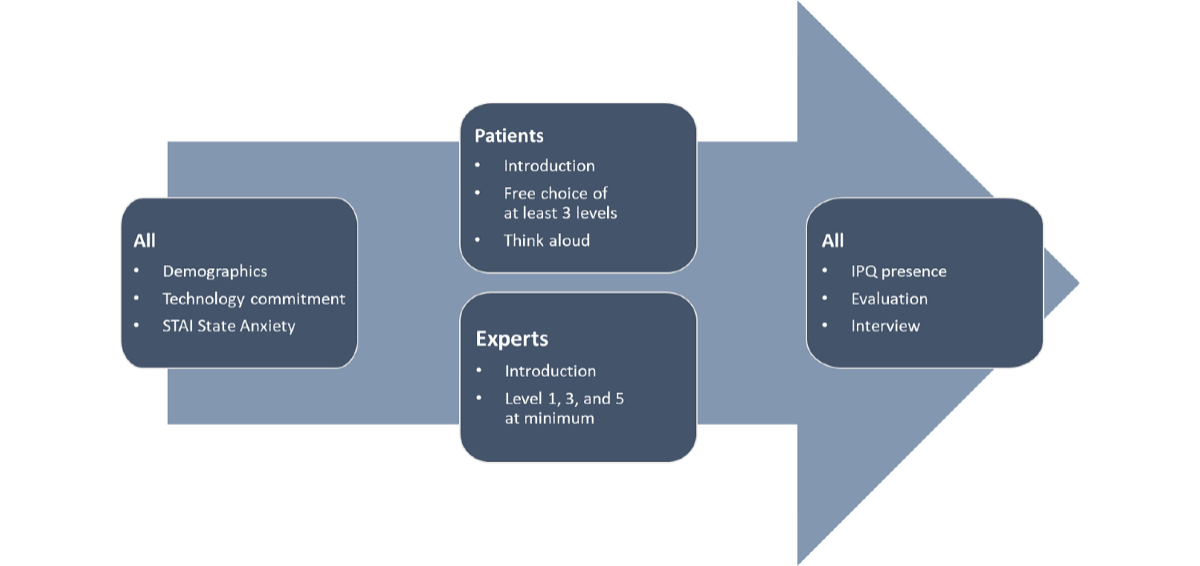
Process of measurements. IPQ: Igroup Presence Questionnaire; STAI: State-Trait Anxiety Inventory.

The semistructured interviews posttreatment included 6 questions that referred to an overall impression of the intervention, the perception of the intensity levels, the assessment of effects of the intervention for patients, and suggestions for improvement. Further questions relating to specific patient groups and implementation prerequisites were asked to the experts only. All questions are presented in Multimedia Appendix 1. The whole process is illustrated in Figure 1.

### Virtual Environment

**Figure 2:**
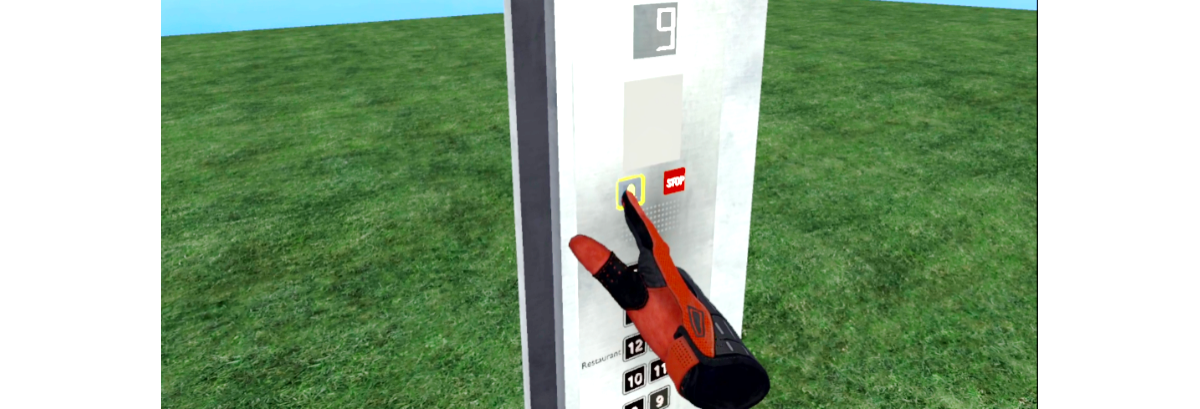
Own hand pressing a button by using the controller during the introduction to the program.

**Figure 3:**
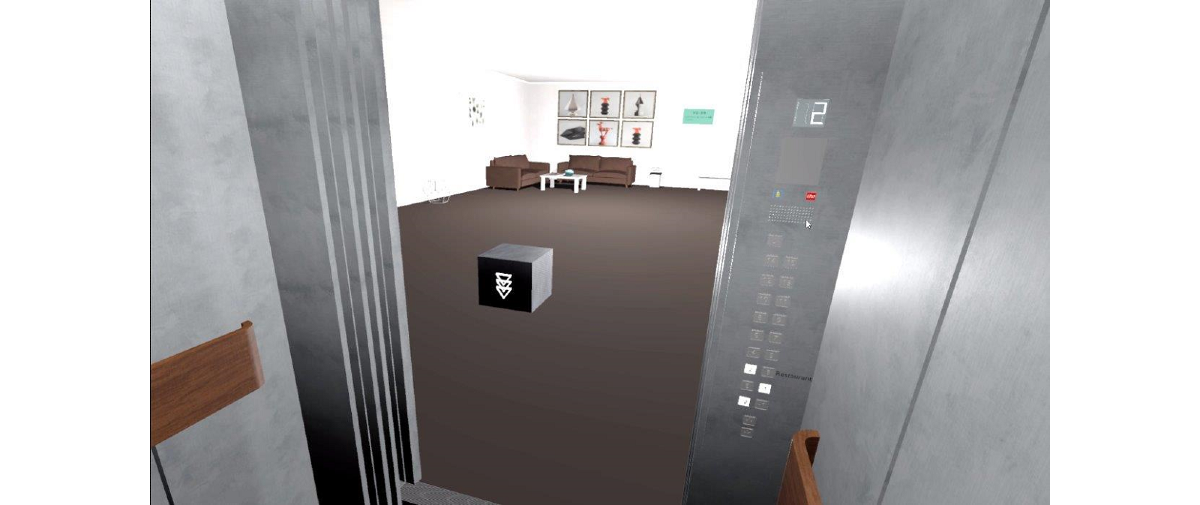
View out of the elevator with the final cube (to be pressed down).

Before starting the virtual tasks, all participants were provided with an introduction by a female voice guiding them through the core functionalities of the program. This was performed using a scenario of the frame of an elevator placed on green grass, without a roof or walls, consisting only of a bottom panel, pillars, and an operating panel of the elevator in order to avoid feelings of claustrophobic anxiety at this stage (Figure 2). The participants were instructed to try out the operating panel and to understand 2 buttons: A yellow alarm clock could be pressed to receive the instruction again. A red “stop” button would immediately finish the level and lead to the main menu in the case of an emerging panic attack. The end of each session was marked by a gray cube that should be pressed downward (Figure 3). During the introduction as well as later during the tasks, the participants were able to see their own hands but not their body.

**Figure 4:**
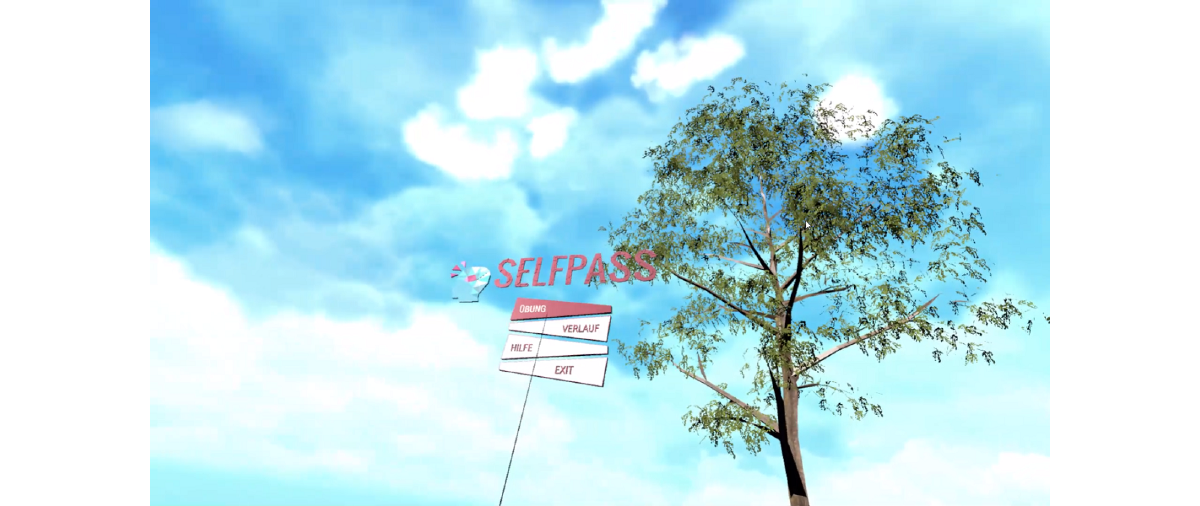
Start screen; after each level, more trees were "won." SELFPASS: Self-administered Psycho-Therapy Systems.

After completion of the virtual introduction, the participants reached the main menu, which was presented in a blue sky over green grass. Here, they were asked to choose 1 of 5 levels by pointing at and clicking a number with their controller. After that, 3 questions were visually and acoustically presented: “How high is your fear or unease when you think about this task?”, “How high will your fear or unease be when you will have completed the task?”, and “How high is your commitment to complete this task?” These questions were presented as 10-point scales from 0 (no fear) to 10 (extreme fear) and as percentages to assess the commitment. After the completion of each task, participants were asked to assess the amount of actual fear felt at that moment and to rate their willingness to perform this task again. After each successful task, the environment changed and trees grew on the green grass as an element of gamification (see Figure 4).

**Figure 5:**
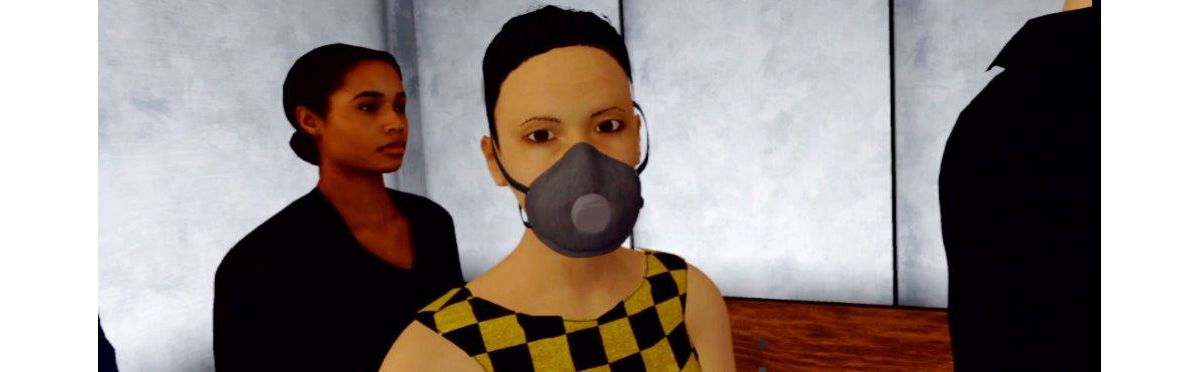
Female virtual passenger at level 4.

**Figure 6:**
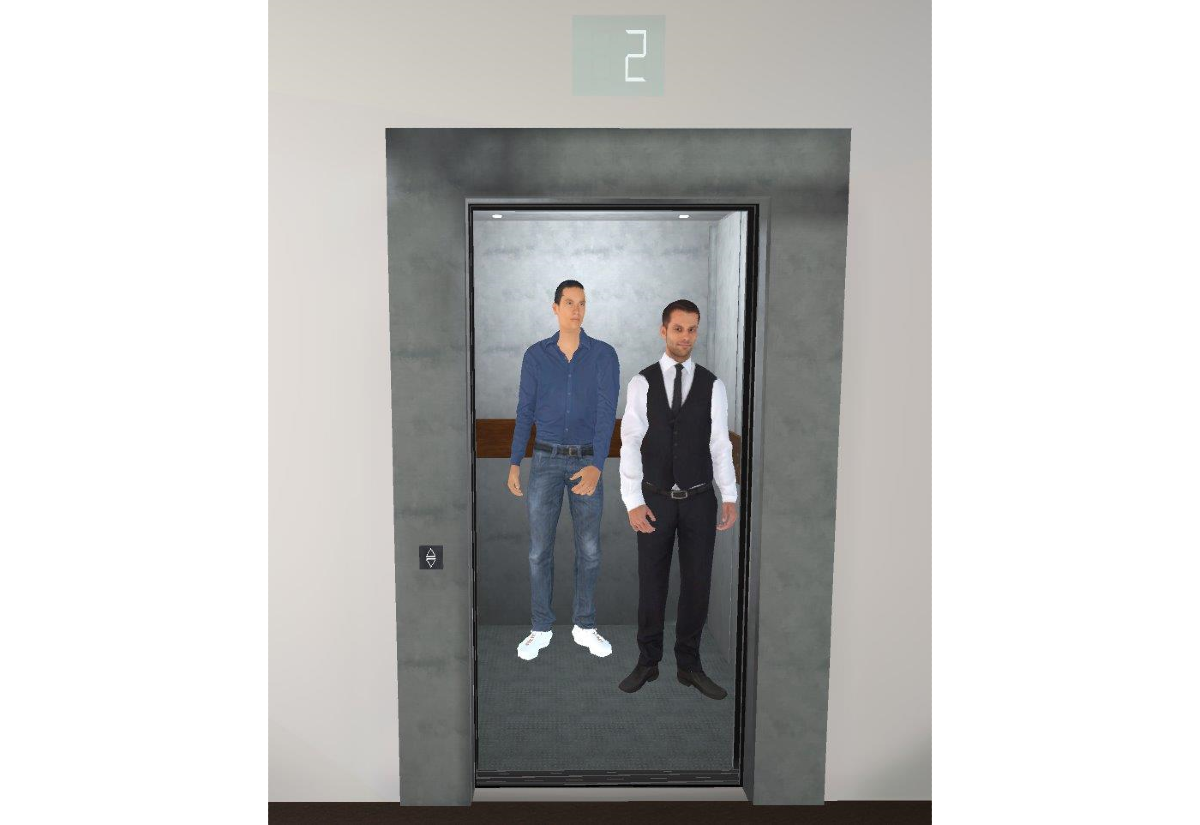
Two male virtual passengers at level 3.

The virtual task was an elevator ride in a simulated office building. Five levels of difficulty with increasing “claustrophobic” challenges were created to graduate the intensity of exposure. Four factors were systematically manipulated to reach the increasing intensity: the size of the elevator, the duration of the ride, brightness, and the number of passengers. The size was varied with 3 sizes. The duration was varied by 3 variables: duration of opening and closing the door, duration to reach the next floor, and duration of stopping at each floor. The virtual passengers were able to act responsively when the participants sought eye contact by returning the gaze, but they were not able to talk. Examples are presented in Figures 5 and 6.

The first level was a simple task: reaching the second floor from the ground floor in a large empty elevator without any passengers (Figure 3). The second level was again a ride in a rather large elevator from the second to the sixth floor, with 1 female passenger already in the elevator. At the third level, 2 male passengers were in an elevator of medium size, leaving less space for the participant who had to go from the office on the 6th floor to the restaurant on the 12th (Figure 6). The fourth level included 3 passengers, among whom 1 female wore a COVID-19 mask and coughed (Figure 5). The size of the elevator was medium. The task was to get from the ninth floor down to the ground floor. At the final level, no passengers were in the elevator, but the task was to get from the parking area on the second floor to the ninth floor. At this stage, the elevator was as small as in none of the previous scenarios, with dark lighting and old elevator noises. With each level, the waiting time in front of the elevator increased, while the speed of the elevator during the ride reduced. In addition, the VR environment allowed participants to get out on another floor, but the level could only be completed in the respective level that had been communicated in the task.

### Data Analysis

The quantitative data were first analyzed descriptively, and means, SDs, frequencies, and percentages are reported. The scores of the 3 scales STAI-S, TCS, and IPQ were calculated according to the manuals. We carried out a reliability analysis for the scales, the STAI-S reached α=.93, the TCS showed α=.87, and the IPQ’s α was .81. Therefore, all scales reached a good level of reliability [61]. The IPQ items were recoded so that the original range of –3 to +3 was transferred to 0 to 6. We explored differences between the groups in the TCS and IPQ scores by *t* tests. In the case of a violation of the homogeneity assumption, we chose the Welch test results. In the case of the STAI, which is usually treated as an ordinal scale [62], we preferred a nonparametric test and performed a Mann-Whitney *U* test [63]. A significance level of *P*<.05 was considered statistically significant. Evaluation items were recoded, if necessary, and an evaluation score from 1 (low evaluation) to 5 (high evaluation) was calculated out of the first 13 items. Two further items referred to the need of assistance by a therapist and to the potential to carry out the intervention alone. These items were calculated separately. Statistical analysis was carried out using the Statistical Package for the Social Sciences (IBM SPSS Statistics ver. 24).

The think-aloud protocols of the patients and the semistructured interviews of all participants were transcribed and analyzed with the help of MAXQDA [64]. The think-aloud protocols were analyzed by 1 of the researchers (author JB) and supervised by author GM. Two coders (authors GM and SH) carried out the analysis of the interviews. The research team thoroughly discussed the 2 coding systems, and disagreements were resolved. Following the rationale of analysis by an inductive approach, as suggested by Mayring [65], new codes were added when new aspects emerged from the data; however, after reaching half of the material, no new codes were accepted. In the qualitative part, we did not count codes, as the frequent occurrence of a topic might not reflect its importance but rather the willingness to talk longer on that topic than on another [66].

## Results

### Participants

A total of 30 participants took part in the study, with 15 (50%) patients and 15 (50%) experts. The mean age of all participants was 40.14 (SD 14.33) years. The patients’ age ranged from 20 to 72 years and showed a mean of 46.07 (SD 17.48) years. The age of the experts ranged from 26 to 41 years, with a mean of 33.79 (SD 17.48) years. Demographic details are presented in Table 1.

**Table 1:** Demographic characteristics of the study sample.

Characteristics		Participants, n (%)		
		Patients (n=15)	Experts (n=15)	All (N=30)
**Gender**				
	Male	8 (53.3)	9 (60.0)	17 (56.7)
	Female	7 (46.7)	6 (40.0)	13 (43.3)
**Profession**				
	Student	2 (13.3)	N/A^a^	N/A
	Employee	3 (20.0)	N/A	N/A
	Retired	4 (26.7)	N/A	N/A
	Not employed	3 (20.0)	N/A	N/A
	Other	3 (20.0)	N/A	N/A
**Therapist background (multiple choice)**				
	Psychodynamic therapy	N/A	9 (60.0)	N/A
	CBT^b^	N/A	5 (33.3)	N/A
	Systemic family therapy	N/A	2 (13.3)	N/A
	Other	N/A	1 (6.7)	N/A

^a^N/A: not applicable.

^b^CBT: cognitive behavioral therapy.

### Pretreatment Scores

Patients showed the highest scores of state anxiety before starting the intervention. A Mann-Whitney *U* test indicated that the anxiety of the patients was higher than that of the experts: *U*=154, *P*=.03. Experts expressed higher scores in technology commitment than patients (see Table 2). The *t* tests showed significant differences in the total score between technology commitment (*t*_28_=2.19, *P*=.04) and technology competence conviction (*t*_28_=3.53, *P*=.002). Other subscores were not significantly different between the 2 groups (technology acceptance: *t*_28_=0.90, *P*=.38; technology control conviction: *t*_28_=1.01, *P*=.32).

**Table 2:** Scores of STAI-S^a^ and TCS^b^ pretreatment.

Scale		Patients (n=15), mean (SD)	Experts (n=15), mean (SD)	All (N=30), mean (SD)	*P* value
STAI-S (N=29)^c^		45.79 (10.46)	36.80 (8.29)	41.14 (10.30)	.03
**TCS**					
	Total	3.74 (0.56)	4.23 (0.67)	3.99 (0.66)	.04
	Technology acceptance	3.17 (0.87)	3.47 (0.96)	3.32 (0.91)	.38
	Technology competence conviction	4.55 (0.91)	5.48 (0.47)	5.02 (0.86)	.002
	Technology control conviction	3.50 (0.45)	3.75 (0.85)	3.63 (0.68)	.32

^a^STAI-S: State-Anxiety Scale of the State-Trait Anxiety Inventory.

^b^TCS: technology commitment scale.

^c^Data of 1 patient were missing for the STAI-S.

### Posttreatment Scores

The VR intervention reached an IPQ presence score of mean 3.84 (SD 0.88), and its highest subscore was the IPQ spatial presence (mean 4.53, SD 1.06).

The *t* tests revealed significant differences in IPQ spatial presence (*t*_28_=2.50, *P*=.02). There was no significant difference between the 2 groups regarding IPQ involvement (*t*_28_=–0.30, *P*=.77), IPQ experienced realism (*t*_28_=–0.71, *P*=.49), and IPQ total (*t*_28_=0.48, *P*=.63).

For details of posttreatment IPQ scores, see Table 3.

Overall evaluation was high (mean 4.25, SD 0.32). Answers to the question “I think such interventions are better conducted in the presence of a therapist” had a mean score of 4.13 (SD 0.83) for patients and 3.33 (SD 1.54) for experts, while the question “I could imagine carrying out such interventions alone in the future” had a mean score of 4.20 (SD 1.01) by the patients and 4.00 (SD 1.07) by the experts. All details of further evaluation items are presented in Multimedia Appendix 2.

**Table 3:** Scores of IPQ^a^ presence (range: 0=lowest, 6=highest).

Scale	Patients (n=15), mean (SD)	Experts (n=15), mean (SD)	All (N=30), mean (SD)	*P* value
IPQ (total)	3.77 (0.91)	3.92 (0.87)	3.84 (0.88)	.63
IPQ spatial presence	4.08 (1.12)	4.97 (0.82)	4.53 (1.06)	.02
IPQ involvement	3.90 (1.25)	3.77 (1.23)	3.83 (1.22)	.77
IPQ experienced realism	2.88 (1.22)	2.62 (0.80)	2.75 (1.02)	.49

^a^IPQ: Igroup Presence Questionnaire.

### Qualitative Results I: Think-Aloud Method

Of the 15 patients, 12 (80%) decided to share their observations and experiences with the think-aloud technique during the intervention, while 3 (20%) made only short comments when technical problems occurred. All codes and subcodes are listed in Table 4. In the following section, the codes “feelings and emotions,“ ”self-assessment,“ and ”telepresence“ are elaborated. The 3 codes ”intensity levels,“ ”own technical expertise,“ and ”technical problems“ are related to comments on the quality of different levels in comparison to other software and to problems during the handling of the program.

**Table 4:** Patients’ expressions during the intervention (think-aloud method).

Codes	Subcodes
Feelings and emotions	fear, tension, relaxation, emotional coping, perception of the virtual humans, perception of the elevator
Self-assessment	anxiety, willingness, motivation, satisfaction
Telepresence	involvement, spatial presence, realism
Intensity levels	N/A^a^
Own technical expertise	N/A
Technical problems	control problems, software errors, interaction with program/supervisor

^a^N/A: not applicable.

#### Feelings and Emotions

A broad category of ”feelings and emotions“ described notions of fear, tension, and relaxation. Moreover, emotional coping was a feeling that was expressed as a positive emotional reaction to a threatening situation. Finally, perceptions of the virtual humans and of the elevator were expressed in a highly emotional way and therefore coded as feelings and emotions as well.

Many patients reported directly feeling claustrophobic symptoms, for example, in the following expression:

Oh God. Woah...why do you build something like that? I always feel like being buried alive.

Patient, 48 years, male, fear/perception of the elevator

The feelings and emotions that patients reported during the intervention revealed that their anxiety could not be summarized as claustrophobic symptoms alone. Due to the presence of 1-3 virtual humans from level 2 on, further symptoms were reported that might be explained by shame or a feeling of not being able to escape from the situation, as observed both in social phobic and in agoraphobic patients.

The two people, one person looked at me, I don't like being watched, it's sort of uncomfortable and then the noises of the person standing behind me,…exactly so the fear was then getting in and driving higher and when I'm out, I can't think of a better term for it, I felt relief there to get out.

Patient, 31 years, male, fear/perception of the virtual humans

*Relaxation* was expressed by patients after completion of a level:

I was happy to just be out and then the thing was, then I could literally leave it behind me.

Patient, 31 years, male, relaxation

Moreover, patients talked about applying *emotional coping* while feeling their symptoms of claustrophobia:

I would like to lean against the wall a bit, but that's not possible right now…Yeah. Wall in the back for sure, because nothing can come from behind…Yes, that gives security that nothing is coming from behind and since I also partly know, I can already tell that, ah ok (yells), I hate it when it counts down and I don't know what's coming out.

Patient, 28 years, male, fear/emotional coping

#### Self-Assessment

Before and after the intervention, the users were asked to assess the degree of anxiety that was expected in advance or experienced retrospectively, the degree of motivation, and the willingness to repeat the intervention. Some patients took this task seriously and answered in a sophisticated way:

How willing are you to repeat this exercise? Since I take it as a learning success, I'll say 80%, 100% would be up here, because I'd like to escape from something like that. But since I just take it as a starting point to further work on my problems I'll just say 80%.

Patient, 28 years, male, motivation/ willingness

Patients often stayed in contact with the psychologist during this part of the intervention. When they chose a level of anxiety felt with their controllers, it was coded as part of the self-assessment and not under the code ”feelings and emotions“:

Well, so also 5-6. I'm shaking already, see?

Patient, 72 years, female, anxiety

#### Telepresence

The patients reported feeling spatial presence, realism, and involvement during the VRE. The code *spatial presence* reflected the perception of the room, while *realism* was given when elements of the intervention tended to be confounded with reality.

To be honest, I'm a bit scared that I'll run into something, hehe.

Patient, 29 years, male, spatial presence

Whoa, isn't it normal? It's not normal that you're afraid of virtual people?

Patient, 48 years, male, realism

However, some of the patients expressed still being aware of the difference between the real and the virtual environment. This awareness provided a feeling of controllability, as shown in the following quote:

The good thing about it is that I know that nothing can happen to me in this case…With a normal elevator, it even starts when I go down a few centimeters...

Patient, 58 years, male, realism

*Involvement*, in turn, indicated that the participants were submerged in the experience.

But now I was so focused on it that I didn't understand which floor, haha. Believe 2nd, but there I am.

Patient, 36 years, female, involvement

### Qualitative Results II: Semistructured Interviews

The analysis of the semistructured interviews posttreatment revealed 8 main categories with 34 subcategories in total (Table 5). In the following section, we report all coding in short and provide examples.

**Table 5:** Codes and subcodes of the qualitative analysis.

Codes	Subcodes
Feelings and emotions	fear, tension, controllability, distress, symptom improvement, positive experiences, perception of virtual humans
Personal story	history of anxiety, personal background
Telepresence	virtuality-reality discrepancy, immersion, realism, perception of the space
Potential therapeutic effects	needs of patients, experiences as a therapist, intensity levels, risks, therapeutic approach, comparison with in vivo, effect
Barriers	financial/technical effort, therapeutic approach
Conditions and requirements	costs, premises, therapeutic setting
Future prospects	therapeutic potential, target group, future challenges
Realization	gamification, hardware, software, suggestions for improvement, personalization

#### Feelings and Emotions

The participants talked about their experiences during the VR intervention in a detailed way and openly shared their personal feelings, such as fear, tension, or distress. *Fear* was observed in both groups. However, experts talked about their anxious feelings in another way (eg, using words such as ”unpleasant“ or ”odd“). Two factors were often reported as triggers for the anxious feelings: the presence of the other virtual humans and the fear of getting stuck:

I know my fears. I asked myself “what happens next?” and “does it get stuck?” That would have been the super disaster. Of course, then you can take off your glasses. However, I would not have done it, for whatever reason.

Patient, 53 years, male, fear/history of anxiety

Moreover, most participants assessed the *perception of virtual humans* as strange and frightening. In particular, the male virtual humans, who were quite tall, caused negative feelings. One of the experts hinted at the possibility of potential risks for female patients with a sexual trauma. One patient even expressed feelings of being negatively concerned with fictitious expectations of the virtual humans:

As I'm in a hotel or something like, um, waiting for an elevator…there is actually such a tension, um, that it could get uncomfortable…And this waiting and then the people in the elevator. You don't want to attract negative attention or anything.

Patient, 29 years, male, tension/perception of virtual humans/history of anxiety

Some participants insisted on keeping control over the situation, as interpreted with *controllability*:

Accordingly, uh, you always have it in your head: Yes, I can also cancel it.

Patient, 53 years, male, controllability/virtuality-reality discrepancy

Many participants reported *positive experiences*. They assessed the VR intervention as appealing and useful. Specifically, patients talked about the individual *symptom improvement* that they observed within themselves after the intervention by passing through stepwise different levels. In this context, participants expressed the wish to have more than 5 intensity levels, which in turn was an important factor for *realization* (see next).

#### Personal Story

As the participants were invited to talk frankly about personal feelings, some of them took the opportunity to talk about their own story, either their *personal background* or their *history of anxiety*. The latter included descriptions of patients that went beyond symptoms of claustrophobia, as most of them were burdened by complex psychosomatic symptom profiles. Some patients, for example, reported having difficulty breathing and were sometimes forced to take an elevator, even if the anxiety is ever-present. Other patients reported to have severe panic attacks that caused them to resort to psychiatric medication.

Some patients were able to develop own strategies of self-efficacy in their past:

I had such terrible panic attacks. And it happened to me in the supermarket and I was so embarrassed, I could,...I never knew how to pay…So I had to face the situation every time. After two or three times I noticed: No, nothing happens in the supermarket.

Patient, 67 years, female, history of anxiety

#### Telepresence

Nearly all participants talked about the degree of telepresence they experienced during the intervention. Their statements referred to the *discrepancy between virtuality and reality,* *immersion*, *realism of the environment*, and *perception of space*, of which all terms will be explained as follows: First, some participants reported feeling remaining doubts regarding the presence of the virtual world, which was termed as *virtuality-reality discrepancy.* The following example illustrates the ambivalence of this experience:

Because there was in the back of your mind, it is now like this...it is not real in the sense that you normally have it. Where you then walk stairs.” “Understand.” “Before you get into the elevator.”

Patient, 58 years, male, virtuality-reality discrepancy

However, some participants said that only the cable reminded them of the existence of the real world, while 1 participant stated that she almost leaned against the elevator wall, as she usually does during an elevator ride. Both examples serve as an illustration for *immersion* as an effectual prerequisite of the feeling of presence.

The *perception of space* was highly influenced by the presence of virtual humans, which seemed to contribute to the limited space in the elevator:

The most difficult for me were the many people. That was the most difficult, yes. Yes, that makes the whole thing even tighter...Because of the tightness because of the people in such a small elevator.

Patient, 58 years, male, anxiety/perception of virtual humans/perception of the space

As a final characteristic subcode within *telepresence*, *realism* described the sense that the design and realization of the intervention was conducted in a realistic way. This issue raised some critical remarks, as the graphical realization of the virtual humans was not assessed to be state-of-the-art technology, and therefore some quotes were categorized as well as *suggestions for improvement* within the main category “realization.”

#### Potential Therapeutic Effects

The participants commented in detail on the perceived usefulness of the VR interventions for therapeutic purposes. *Patients' needs* were in the focus of strong concerns, as many participants observed that the VR intervention might not be suitable for every kind of patient. Elderly patients might not benefit from this kind of technology; moreover, some patients could need more individual cues to trigger their anxiety, and therefore, a broader range of intensity levels is needed. The latter directly refers to the subcode *intensity levels,* which were assessed as not fully building up on each other in terms of difficulty. The final intensity level took place at night and alone in a small elevator, whereas levels 3 and 4 included virtual humans. Many participants, but not all of them, found the sessions with the virtual humans more difficult than being completely alone at level 5.

The experts argued from their professional perspective when talking about their *experiences as therapists.* In doing so, they voiced the wish for controlling the situation and the time frame, as it might be necessary to stay for a long time in a situation until the anxiety decreases. The therapists appreciated the possibility of a smooth start for patients and assessed VRE as *comparable to in vivo* or as a starting point:

I can also imagine that it will be well accepted, because you can't make a fool of yourself in that sense…It's just something different when you really go to the department store and do some exercise.

Expert, 40 years, male, therapeutic potential/comparison with in vivo

Finally, *related risks* raised by the participants mainly referred to a potential scenario of doing the VRE alone without guidance. In this case, some participants identified an increased danger of being left alone with a panic attack.

#### Barriers

There were a few reasons participants would not use the VR intervention for therapeutic purposes. These reasons can be summarized by restrictions due to high *financial* or *technical efforts* that were seen by those therapists who were talking about therapy in a private practice, where care is not provided by a clinic. Other barriers were reported by therapists who followed other *therapeutic approaches* than CBT. Especially psychodynamic therapists stated not to concentrate on a mere behavioral training when treating an anxiety disorder, as expressed, for example, in the following statement:

And with whom I wouldn't do it? I don't think it will help those who—I think—organize or express their social needs by their fears.

Expert, 30 years, female

#### Conditions and Requirements

The category “conditions and requirements” showed some overlapping meanings with “barriers”; however, additional aspects, such as requirements and practical considerations, were reported in this context. Subcodes included *cost*, *facilities*, and *therapeutic setting*. Many participants, especially experts, but also patients, considered the presence of a face-to-face therapist as important for the success of the VR intervention:

In my eyes, the introduction…is actually very well suited to reduce this fear and to be able to get involved with this whole thing once. And therefore…it is definitely important to include other people to support, from a therapeutic perspective, definitely.

Patient, 31 years, male

Nevertheless, some of the experts were convinced that patients should use the intervention as mere self-management training at home:

So that you can say this has to be practiced now, another thing has to be practiced next week…of course, people must have a setup. Well, I don't feel like practicing it in a therapy session, I think so, really, but I would like to let people do it at home.

Expert, 41 years, male

Experts who were in favor of a guided setting expressed a desire for the features to be directly controlled by the therapist during the session (eg, the degree of narrowness, the number of virtual humans). This aspect is strongly related to features of personalization, which were also included in “realization.”

#### Future Prospects

Overall, the participants described some future prospects for this kind of VR intervention. They saw much *therapeutic potential* in the scenario for different ways of treatment (eg, by addressing more fears than just claustrophobia, by adding a glass floor for patients with acrophobia). Further, they defined *target groups* that were more or less suitable for comparable VR interventions. Future challenges were seen in the successful integration into clinical practice; however, many technological prospects might allow new possibilities of treatment, as 1 expert elaborated:

I thought about whether you could still build in wearables, there are so many watches that measure the pulse, for example if you could somehow integrate it into the system that you have markers like biofeedback. I would think that's great because, I mean this, shall we say, physiological stress reaction that you have that always stops. So if you are, let's say you are really claustrophobic and you stand in the elevator with all the people, then you have, you are in a panic state that lasts for a long time but at some point it stops and that's that what you show people over and over again with biofeedback.

Expert, 40 years, male

#### Realization

Technical realization was assessed in a differentiated way by the participants. The *gamification* elements after each level were much appreciated. *Hardware* and *software* were seen as leaving room for future improvements, either by finding a solution without the annoying cable on the HMD or by delivering a more stable connection, as glitches and distortions were reported in some cases. However, none of the participants reported feeling motion sickness, which is a frequently felt consequence of VR interventions.

Suggestions for improvements were collected as well. They included ideas for more elaborated intensity levels that should be directly controllable by the therapist.

The participants recommended providing more opportunities for personalization features, not only delivered by the therapist, but also delivered for the patients themselves:

What I still find cool would-be customizability, no, that is, that you create a personal fear hierarchy and then maybe adjust the levels accordingly.

Expert, 30 years, female

## Discussion

### Principal Findings

Although the effectiveness of VRE in the treatment of anxiety disorders is well studied and the comparability to in vivo trainings could be shown in numerous studies [9,14,18,67], to date, few studies have examined the experiences of patients in the case of VRE for claustrophobic symptoms. This study used a mixed methods approach to ask for the experiences and perspectives of patients and therapeutic experts testing a VRE app with different intensity levels for claustrophobia with the use of additional virtual humans within the environment. Our research intended to understand the inner processes of patients with anxiety during VRE sessions in order to define key elements necessary for an effective exposure setting. The perspective of therapeutic experts was added to understand potential facilitators and barriers, as well as professionals' view on the target group and treatment effects. In the long term, the results might serve to improve the design of comparable apps.

In our results, patients, who initially had the highest pretreatment anxiety, scored lower than the experts in the total presence score of the IPQ and in the subscore IPQ spatial presence but higher in IPQ involvement and IPQ experienced realism; however, the difference was only significant in the case of spatial presence. In addition, the qualitative interviews revealed that a considerable proportion of participants felt the discrepancy between reality and virtuality (eg, by feeling the cable in their back). Both groups gave high ratings in the evaluation of feasibility and acceptability, and the scores were higher in the patient group.

The patients were asked to think aloud while carrying out the intervention, and their feelings of anxiety and tension support the assumption that the intervention successfully led to the desired effect. However, some symptoms were closely related to the presence of 1-3 virtual humans from the second intensity level on. These virtual humans were included to make the intervention more realistic and provide a further feeling of narrowness, but they may have evoked sociophobic or agoraphobic symptoms as well. Specific phobias and agoraphobia show a correlation of r=0.57 in the literature [68]; however, studies that report comorbidities specifically between claustrophobia and other anxiety-related disorders are missing. Further results could be derived from semistructured interviews with the participants. These results repeated the quantitative results, as feelings of presence and involvement were reported in both groups.

Recommendations for the methodology of VR clinical trials in health care were recently provided by an international working group. Birckhead et al [69] recommend 3 types of VR trials: VR1 studies for content development, VR2 studies for proof of concept, and VR3 studies for clinical evidence. Our study followed the rationale of VR2 studies. For those studies, the authors suggest investigating the following parameters: patient population, clinical setting, assessment of acceptability, feasibility and tolerability, and, finally, assessment of initial clinical efficacy. The latter was conceptualized as patient-reported outcomes, as objective clinical outcomes are recommended for randomized clinical trials (RCTs) only [69].

Following the criteria of Birckhead et al [69], the *patient population* was assessed by targeted recruiting, including SCID interviews, and supported by the results of the STAI-S, which exceeded the cut-off of 41 in the patient group [57]. Moreover, technology commitment was assessed in advance, and even though patients showed the lowest commitment, the 2 groups stayed comparable to the population sample of the scale developers [56]. Low levels of the total score of technology acceptance of patients might be explained by the lower score of technology competence conviction in comparison to the experts. This, in turn, might be due to the higher age of some of the patients and should be considered in future clinical use. The *clinical setting* was established as the intervention was tested within the facilities of the Heidelberg University Hospital and with the guidance of a trained psychologist. *Feasibility* and *acceptability* were assessed by evaluation items and by the semistructured interviews; the results indicate that both may be considered to have been reached. However, some experts remained skeptical, especially when they followed a psychodynamic therapy concept. Unlike cognitive behavioral therapists, psychodynamic approaches often aim at emotional experience rather than at habituating high levels of anxiety aroused by a situation [70]. *Tolerability* was assessed qualitatively, as the participants got an opportunity to report on their own experiences during the intervention. None of the participants had to stop the intervention due to feeling overwhelmed or motion sickness, which is a frequent limitation in the usage of VR apps [71]. The *initial clinical efficacy* was supported by the qualitative statements and could be shown as patients reported personal success in their self-assessments. However, further sessions with time intervals between sessions would be necessary to prove efficacy.

### Therapeutic Setting of VRE

An ambivalent topic was the question of whether the intervention is an exercise that can be conducted at home or whether the physical presence of a therapist is necessary. It has to be stated that therapeutic guidelines recommend that a therapist stay by the side of the patient [7]. On the contrary, there is growing evidence that a self-management app for VRE leads to symptom reduction and is at least not inferior to that with therapist guidance [45,72]. Hence, both kinds of apps are used in practice [73]. In Germany, there is a self-management app for anxiety with integrated VR training that is covered by health insurance [74]. However, therapists might miss the opportunity to interact with the patient during the intervention and control variables (eg choice of stimuli, duration, or intensity levels) [14].

In our results, the patients scored high on the question of whether the intervention should be accompanied by a therapist and at the same time they were also convinced that the intervention could be conducted alone at home. These ambivalent results may reflect the uncertainty toward the new technology. However, future studies are necessary to support our interpretation. With regard to in vivo treatment, we recommend that the first sessions be always conducted in the presence of a therapist who might then decide whether the patient is able to undergo the training alone at home.

In fact, the uptake of VR interventions in clinical practice remains hesitant [75]. Our results have shown that although most of our experts, regardless of their therapeutic background, showed positive attitudes toward the intervention, barriers might arise due ot costs and the need for a separate room. Another limiting factor seems to be a lack of evidence-based software that can be purchased and integrated into one's IT facilities. Either the software is available but not clinically tested, or the software is developed within a scientific infrastructure, well tested, but not commercially available [17].

Based on our results, we suggest the following key elements for a successful virtual exposure:

- Provide context variation by varying relevant factors regarding size, duration of a setting, and increasing darkness [10].
- Allow for systematic variation of the factors in order to provide an opportunity for individualized training.
- Add self-assessments within the treatment before and after a session regarding the respective anxiety level actually felt by the patient.
- Include virtual humans that should be personalized to the needs of the patients with respect to comorbidities (social phobia, traumatic experiences).
- Provide a safe therapeutic setting for exposure, as recommended by treatment guidelines [7].

### Directions of Future Research

The potential of VRE might be enhanced by the possibility to add objective data to the personal feelings of the patient by integrating sensor data collected with a wearable wristband. These results can lead to personalized suggestions for the respective adequate intensity level. With the integration of real-time physiological data, a validation of the prerequisites of effective treatment might be possible, as former studies have shown that different scenarios in film, text, or VR induce different patterns of parasympathetic activation, with the lowest result in VR despite the highest self-reported presence [76]. This result is in line with the statement of Böhnlein et al [10], who found that the avoidance of relaxation is important for the success of VRE. Finally, future directions must meet the growing demands of personalized digital interventions. For example, the sex of the virtual humans as well as their age and culture-specific features should be tailored to the user [77].

### Limitations

To the best of our knowledge, this is the first study to examine the experiences of patients with anxiety testing a VRE app for claustrophobia with virtual humans and made use of the think-aloud method with this target group. However, some limitations could not be avoided. First, claustrophobia is a seldom-given diagnosis, so we relied on self-reported symptoms of patients with a diagnosis of any anxiety disorder. Therefore, some experiences and feelings reported by the patients might be due to other fears and not just claustrophobia. As mentioned in the introduction, some authors already stated that claustrophobia can be understood as a prodromal stage of agoraphobia [43]. Second, we only measured state anxiety before starting the intervention, and therefore, an assessment of clinical efficacy was only conducted qualitatively and should be repeated with a longitudinal design, including more differentiated measures to assess the aspects of claustrophobia (eg, fear of suffocation and of confinement). Third, our anxiety assessment during the intervention was only implemented as a beta version. Future implementation will allow for data export for therapists to see the patients' progress. Fourth, although the think-aloud methodology is a renowned method to get in touch with the immediate feelings and emotions of a person, it might impede the process of immersion and presence. Future study designs need to find a way to resolve this paradox. Moreover, the fourth intensity level of our intervention included a virtual coughing woman wearing a protective mask. By this, we wanted to ensure a high degree of realism; however, it cannot be excluded that health-related fears may interfere with our results. Finally, the participants could choose an individual level of intensity. Future studies will investigate the exact effects of all levels with a higher number of participants in an experimental design with systematic variation.

### Conclusion

A VRE app for claustrophobia with different intensity levels and with the presence of a gradually increasing number of virtual humans is feasible for inducing the desired degree of anxiety in patients in order to work with those fears during a therapy session. Key elements of a VRE app for claustrophobic symptoms should provide variation of intensity by adding challenging cues in order to induce presence, which is a necessary state for inducing anxiety. Virtual humans can be included to make the intervention realistic and to provide a sense of closeness; however, some of the fears might then as well be caused by social phobia or agoraphobia. Patients may need the physical presence of a therapist, even if psychotherapists argue that the intervention might be conducted alone as well. More intensity levels are needed, with the option to adapt the intervention to a personalized symptom profile. By doing this, a more specific support might be provided.

## Appendix

Multimedia Appendix 1 Interview questions.

Multimedia Appendix 2 Results of the evaluation of patients and experts (means and SDs).

## Abbreviations

CBT: cognitive behavioral therapy
ET: exposure therapy
HMD: head-mounted display
IPQ: Igroup Presence Questionnaire
SCID: Structured Clinical Interview for DSM-5
STAI-S: State-Anxiety Scale of the State-Trait Anxiety Inventory
TCS: technology commitment scale
VR: virtual reality
VRE: virtual reality exposure
