# Supplementary material for: Experiences of Patients and Therapists Testing a Virtual Reality Exposure App for Symptoms of Claustrophobia: Mixed Methods Study
Source: JMIR Ment Health. 2022 Dec 5;9(12):e40056. doi: 10.2196/40056 (PMC9764154; doi:10.2196/40056)
Supplement: Multimedia Appendix 1 [file mental_v9i12e40056_app1.docx]

**Multimedia Appendix 1**

**Semi-structured Interview**

What is your overall impression of the application? (all groups)

How do you rate the difficulty of the intensity levels you tested? (all groups)

What could be improved? (all groups)

What would prevent you from using the application? (all groups)

Do you think a patient with an anxiety disorder can benefit from the intervention? (all groups)

Are there special patient groups for whom you find the application more or less suitable? (experts only)

What are the prerequisites for implementing the technology in the supply? Where do you see obstacles or risks? (experts only)

Is there anything else you would like to give us feedback that has not appeared in the previous questions? (all groups)

What is your therapeutic specialty? (experts only)

How many years of professional experience do you have? (experts only)
