# Supplementary material for: Experiences of Patients and Therapists Testing a Virtual Reality Exposure App for Symptoms of Claustrophobia: Mixed Methods Study
Source: JMIR Ment Health. 2022 Dec 5;9(12):e40056. doi: 10.2196/40056 (PMC9764154; doi:10.2196/40056)
Supplement: Multimedia Appendix 2 [file mental_v9i12e40056_app2.docx]

|  |  | **Patients  (N=15)**  **Mean (SD)** | **Expert**  **(N=15)**  **Mean (SD)** | **Total (N=30)**  **Mean (SD)** |
| --- | --- | --- | --- | --- |
| 1 | The questions were expressed in an understandable way. | 4.87 (0.35) | 4.67 (0.49) | 4.77 (0.43) |
| 2 | The usage was uncomplicated. | 4.27 (1.16) | 3.93 (0.96) | 4.10 (1.06) |
| 3 | The response of the program was clear and transparent. | 4.80 (0.41) | 4.33 (0.72) | 4.57 (0.63) |
| 4 | I felt taken seriously by the questions. | 4.67 (0.62) | 4.33 (0.90) | 4.50 (0.78) |
| 5 | I think the intervention was too brief (recoded). | 2.73 (1.33) | 3.13 (1.06) | 2.93 (1.20) |
| 6 | I think that the intervention was too lengthy (recoded). | 1.60 (0.83) | 1.47 (0.52) | 1.53 (0.68) |
| 7 | The intervention was structured in an understandable manner. | 4.67 (1.05) | 4.60 (0.51) | 4.63 (0.81) |
| 8 | The language of the intervention was easy to understand. | 4.53 (1.06) | 4.73 (0.46) | 4.63 (0.81) |
| 9 | The intervention bored me (recoded). | 1.20 (0.41) | 2.20 (0.94) | 1.70 (0.88) |
| 10 | The intervention is helpful to overcome an anxiety disorder. | 3.80 (1.37) | 3.80 (1.15) | 3.80 (1.24) |
| 11 | I was able to navigate easily through the program. | 4.47 (0.74) | 4.33 (0.49) | 4.40 (0.62) |
| 12 | The design of the program was appropriate for that subject. | 4.07 (0.80) | 4.27 (0.59) | 4.17 (0.70) |
| 13 | The design of the intensity steps was appropriate. | 4.33 (0.62) | 3.47 (0.83) | 3.90 (0.84) |
| 14 | I could imagine doing such interventions on my own in the future. | 4.20 (1.01) | 4.00 (1.07) | 4.10 (1.03) |
| 15 | I think that such interventions should only be done in attendance of a therapist. | 4.13 (0.83) | 3.33 (1.54) | 3.73 (1.28) |
|  | **Total Score (1-13)** | **4.38 (0.32)** | **4.13 (0.27)** | **4.25 (0.32)** |

Answers of patients and experts to evaluation items (1=”does not apply at all”, 5=”applies fully”; means and standard deviations)
